# Structural Volumetric Alterations in Parkinson's Disease With Mild Cognitive Impairment

**DOI:** 10.1002/brb3.71410

**Published:** 2026-04-27

**Authors:** Jinhua Hu, Peng Lei, Jupeng Zhang, Qi Wu, Zhihao Zhang, Xiqi Zhu, Baosheng Li

**Affiliations:** ^1^ Department of Radiology Affiliated Hospital of Youjiang Medical University for Nationalities Baise China; ^2^ School of Medical Laboratory Science Affiliated Hospital of Youjiang Medical University for Nationalities Baise China; ^3^ Guangxi Engineering Research Center for Precise Genetic Testing of Long‐dwelling Nationalities, Guangxi, China Baise China

**Keywords:** meta‐analysis, mild cognitive impairment, Parkinson's disease, structural volume, subcortical atrophy

## Abstract

**Background:**

Parkinson's disease with mild cognitive impairment (PD‐MCI) is a critical dementia prodrome, but its structural neuropathology remains incompletely defined. While hippocampal atrophy is established, volumetric changes in other subcortical structures are poorly characterized.

**Objective:**

To perform the first comprehensive meta‐analysis quantifying gray matter volume alterations across six subcortical regions in PD‐MCI.

**Methods:**

A systematic search of PubMed, Web of Science, Embase, and Cochrane (inception‐June 2025) for studies reporting MRI volumetric data comparing PD‐MCI and cognitively normal PD (PD‐NC) was done. Random‐effects models calculated pooled weighted mean differences (WMDs) with 95% confidence intervals (CIs). Heterogeneity (*I* ^2^), publication bias, sensitivity analyses, and meta‐regression were assessed.

**Results:**

PD‐MCI showed significant bilateral atrophy versus PD‐NC in the hippocampus (total WMD = –0.65 cm^3^), thalamus, putamen, and amygdala, alongside right‐lateralized globus pallidus atrophy (WMD = –0.08 cm^3^). Bilateral caudate nuclei volumes were preserved. Sensitivity analyses confirmed robustness. Meta‐regression identified segmentation tools and country as sources of left hippocampal heterogeneity (*p* < 0.05). No publication bias was detected.

**Conclusion:**

PD‐MCI exhibits a distinct subcortical atrophy signature involving limbic–striato‐thalamic networks, with right globus pallidus atrophy as a novel lateralized biomarker. Network‐based imaging paradigms are advocated, requiring standardized protocols and longitudinal validation.

**Systematic Review Registration:**

Identifier PROSPERO (CRD420251051275).

## Graphical abstract

This meta‐analysis reveals a distinct subcortical atrophy signature in Parkinson's disease with mild cognitive impairment (PD‐MCI), including bilateral hippocampal, thalamic, putamen, and amygdala atrophy, along with right‐lateralized globus pallidus atrophy, while caudate volumes are preserved. These findings highlight right globus pallidus atrophy as a novel lateralized biomarker and support network‐based imaging approaches.

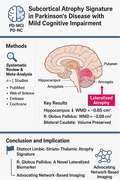

## 1 Introduction

Parkinson's disease (PD) ranks as the second most prevalent neurodegenerative disorder, afflicting over 10 million people across the globe (McKinley and Perkins 2019). PD results from reduced dopamine transmission in the striatal motor circuit, dysregulating the direct and indirect pathways (Latif et al. 2021). Beyond cardinal motor manifestations including bradykinesia, rigidity, and tremor, cognitive dysfunction is a hallmark feature of PD (Aarsland et al. 2021). Current research indicates that early‐stage PD patients with cognitive impairment exhibit white matter and gray matter lesions (Koshimori et al. 2015). Clinically, the Montreal Cognitive Assessment (MoCA) serves as the primary tool for diagnosing and evaluating treatment efficacy. Among the various complex symptoms, cognitive impairment profoundly impacts patients' quality of life and prognosis (Bode et al. 2024). Research shows that approximately 80% of patients diagnosed with Parkinson's disease with mild cognitive impairment (PD‐MCI) progress to Parkinson's disease dementia (PDD) (Goldman and Sieg 2020; Hely et al. 2008). This high conversion rate emphasizes the urgency of early identification and intervention targeting cognitive dysfunction in PD.

The hippocampus, essential for learning, memory consolidation, and spatial navigation, is a critical neuroimaging biomarker of cognitive decline in PD (de Schipper et al. 2019). Subcortical gray matter structures—notably the thalamus, putamen, caudate nucleus, amygdala, and globus pallidus—critically contribute to PD‐MCI pathogenesis (Filippi et al. 2020; Guttuso et al. 2022; Hanganu et al. 2014). The thalamus acts not only as a relay for motor information but also integrates cognitive, motivational, and emotional functions; its dysfunction is closely linked to PD symptomatology (Carvalho de Abreu et al. 2024; Güllüoğlu et al. 2023). Together, these regions constitute the neuroanatomical substrate for PD‐related cognitive impairment: Hippocampal atrophy directly correlates with memory deficits. The putamen, caudate nucleus (head), and globus pallidus are core components of basal ganglia–thalamocortical circuits; their degeneration drives motor symptoms and contributes to cognitive deficits via prefrontal–striatal (caudate) and limbic–cognitive (globus pallidus) pathways (Bocchetta et al. 2021; Drummond and Chen 2020; Oh et al. 2024; Weintraub and Zaghloul 2013). Thalamic atrophy disrupts multiple cognitive domains due to its role in sensorimotor and higher‐order information processing (Choi et al. 2022), while amygdala pathology associates with emotional processing disturbances (Gläscher and Adolphs 2003). Therefore, concurrent degeneration across these regions may underlie the multi‐domain cognitive impairment in PD‐MCI.

Prior meta‐analysis has predominantly examined hippocampal atrophy (Yazdan Panah et al. 2023). However, a comprehensive analysis of volume alterations across key subcortical structures in PD‐MCI patients remains lacking. Expanding beyond prior meta‐analysis confined to the hippocampus, this study quantitatively assesses volume changes in six key brain regions—the hippocampus, thalamus, putamen, caudate nucleus, amygdala, and globus pallidus—in PD‐MCI, thereby elucidating the association between cognitive decline and integrated cognitive–limbic–motor network pathology in PD.

## 2 Methods

### 2.1 Literature Search Strategy

According to the PRISMA guidelines, a systematic literature search was conducted by two independent researchers (HJ and LP). From the inception of the databases to June 19, 2025, data were extracted using standardized forms (PubMed, Web of Science, Embase, Cochrane) using MeSH terms and keywords: Parkinson's disease, cognitive impairment, gray matter, magnetic resonance imaging, volume, atrophy, hippocampus, thalamus, basal ganglia. The titles and abstracts of the retrieved records were screened to meet the inclusion criteria. This study has been prospectively registered on PROSPERO (CRD420251051275).

### 2.2 Inclusion and Exclusion Criteria

Inclusion criteria are as follows: Study subjects: (1) Study design type: original observational study method, with full text available for review. (2) Participants: Patients with PD, further classified as with PD‐MCI or PD‐NC. (3) Volumetric data for at least one of the following structures reported based on MRI measurements: hippocampus, thalamus, putamen, caudate nucleus, amygdala, and globus pallidus. (4) Original studies with no missing data. Exclusion criteria were as follows: (1) Duplicate literature or no full text available. (2) Non‐observational trials. (3) Studies with missing or incorrect data that could not be completed or corrected. (4) Lack of outcome measures required for this meta‐analysis; (5) Letters, case reports, reviews, and practice guidelines. (6) Studies including subjects with MCI or PD who had other significant underlying neurological or psychiatric conditions (e.g., stroke, major depression, other dementias). (7) All animal experiments.

### 2.3 Data Extraction

Two independent investigators (HJ and LP) extracted the data using standardized forms. The extracted information included: publication details (authors, year), research design, participant characteristics (sample size per group, age, sex), diagnostic criteria for PD‐MCI magnetic resonance imaging acquisition parameters (scanner field strength, sequence), segmentation methods (software/tool), and volume data of all reported structures (mean ± standard deviation), as well as details of the structural lateralization. Any discrepancies were resolved through consensus or consultation with a third reviewer (ZX).

Disease duration was extracted when reported. However, due to heterogeneous and incomplete reporting across studies, the available data lacked sufficient granularity to standardize volumetric differences by disease duration, perform disease‐duration meta‐regression, or compute annualized atrophy rates.

### 2.4 Quality Evaluation

Quality appraisal was performed independently by HJ and LP via the Newcastle‐Ottawa Scale (NOS), featuring eight evaluation items within three domains. The NOS assesses study quality across three domains: selection of the study groups (4 items), comparability of the groups (1 item), and ascertainment of the outcome (3 items). A star system is used, with a maximum score of 9 stars. Each item is awarded a star if fulfilled, leading to a total score ranging from 0 to 9 stars. A higher composite score indicates a higher level of research quality. Studies scoring 6–9 points are rated as “high quality,” while studies scoring 0–5 points are classified as “low quality.” The two researchers independently scored the trials, and in cases of disagreement, they reached a consensus or sought the decision of a third‐party evaluator (ZX).

### 2.5 Statistical Analysis

Meta‐analysis was performed using Stata/SE 15.0 (StataCorp LP, College Station, TX). Volumetric differences between PD‐MCI and PD‐NC groups were expressed as Weighted Mean Differences (WMD) with 95% Confidence Intervals (CIs). Volumes initially reported in mm^3^ were converted to cm^3^ (by dividing by 1000). Median and IQR data were converted to mean ± SD using Hozo et al.’s method (Hozo et al. 2005). Heterogeneity was assessed using the *I* ^2^ statistic, with *I* ^2^ > 50% indicating substantial heterogeneity. The DerSimonian–Laird random‐effects model was employed to pool effect sizes when *I* ^2^ > 50%; otherwise, a fixed‐effects model was used. Meta‐regression analysis explored potential sources of heterogeneity (e.g., segmentation tool, MRI field strength, mean age) for hippocampal volume. For regions with significant sources of heterogeneity (e.g., the left hippocampus), group analysis was conducted using different segmentation tools (FreeSurfer and other segmentation tools).

Publication bias was evaluated using funnel plots, supplemented by Begg's rank correlation test and Egger's linear regression test. Sensitivity analysis assessed the robustness of pooled estimates using the “leave‐one‐out” method. Analysis was stratified by hemisphere, where data permitted; bilateral averages were used for studies reporting only whole‐structure volumes.

## 3 Results

### 3.1 Literature Search and Screening Results

**FIGURE 1:**
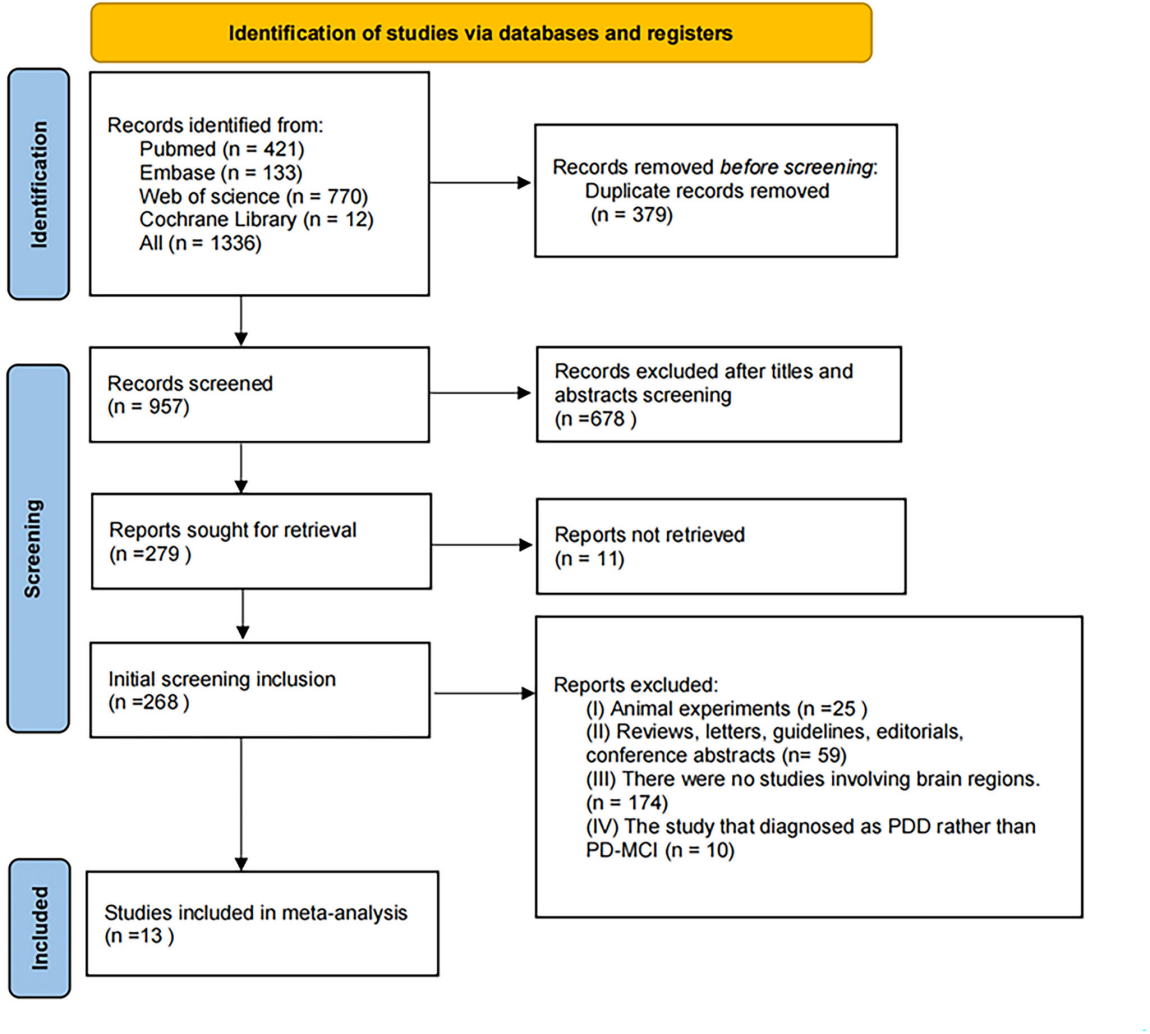
PRISMA flowchart of study selection. PDD = Parkinson's disease dementia; PD‐MCI = Parkinson's disease with mild cognitive impairment. The diagram outlines screening, exclusion reasons, and final included studies (*n* = 13) for meta‐analysis.

The initial search yielded 1336 records. After removing duplicates and screening titles or abstracts, 268 full‐text articles were assessed. Thirteen studies met the inclusion criteria, comprising data from 267 PD‐MCI patients and 387 PD‐NC controls (Apostolova et al. 2010; Dalaker et al. 2011; Danti et al. 2015; Erhardt et al. 2023; Foo et al. 2017; Hunerli‐Gunduz et al. 2023; Hünerli et al. 2019; Kandiah et al. 2014; Legault‐Denis et al. 2024; Mak et al. 2014; Mak et al. 2015; Subotic et al. 2024; Yildiz et al. 2015). The PRISMA flow diagram detailing the selection process is presented in Figure 1. Key characteristics of the included studies are summarized in Tables 1 and S1.

**TABLE 1:** Characteristics of included studies.

Author (year)	Country	Study design	PD‐MCI		PD‐NC		Vendor	MRI sequence	Volume correction	Segmentation tool	PD diagnostic criteria	Cognitive assessment tool
			Sample size (total/f/m)	Age	Sample size (total/f/m)	Age						
YILDIZ 2015	Turkey	Cross‐sectional study	19/NR/NR	59 ± 10	14/NR/NR	55 ± 10	NR	3D MP‐RAGE	Corrected volume	Manual segmentation	UPDRS	MMSE
Subotic 2023	Canada	Cohort	18 (3f/15m)	70.1 ± 8.1	42 (23f/19m)	66.6 ± 7.1	3.0 T GE	3D‐T1, T2, FLAIR	uncorrected	FreeSurfer	MDS‐UPDRS III	MoCA
Mak 2015	UK	Cross‐sectional study	39 (10f/29m)	69.4 ± 8.8	66 (25f/41m)	62.9 ± 9.9	3 T Philips	T1, 3D MPRAGE	normalized	FreeSurfer	UPDRS III	MoCA
E. Mak 2014	Singapore	Cross‐sectional study	25 (7f/18m)	69.4 ± 6.4	65 (19f/46m)	63.4 ± 7.6	3 T Philips	3D T1	normalized	FreeSurfer	UPDRS	NINDS criteria
Legault‐Denis 2024	Canada	Cohort	6 (6f/0m)	68.7 ± 6.5	6 (3f/3m)	65.3 ± 6.7	3T Siemens	T1, 3D MPRAGE	TIV Correction	MAGe T‐Brain	NR	MoCA, MDRS‐2
Kandiah 2014	Singapore	Prospective longitudinal	34 (4f/30m)	67.10 ± 7.22	44 (14f/30m)	62.68 ± 7.54	3 T Philips	T1, FLAIR	corrected	FIRST	NINCDS criteria	MoCA, MMSE
Hunerli‐Gunduz 2023	Turkey	Cross‐sectional study	36 (9f/27m)	68.39 ± 7.13	32 (7f/25m)	66.69 ± 9.27	1.5 T Philips	3D T1	normalized	FIRST	UPDRS	MMSE
Hunerli 2019	Turkey	Cross‐sectional study	20 (5f/16m)	69.86 ± 5.78	21 (4f/19m)	67.30 ± 8.91	1.5 T Philips	3D T1, T2	corrected	FIRST	UPDRS	MoCA, MMSE

Author (year)	Country	Study design	PD‐MCI		PD‐NC		Vendor	MRI sequence	Volume correction	Segmentation tool	Definition of PD/Cognitive assessment	Cognitive assessment tool
			Sample size (total/f/m)	Age	Sample size (total/f/m)	Age						
Foo 2017	Singapore	Longitudinal study	11 (5f/6m)	69.45 ± 10.19	54 (13f/41m)	63.39 ± 6.86	3.0 T GE	T1	corrected	FreeSurfer	Hoehn and Yahr stage	MoCA, MMSE
Erhardt 2023	USA	Cohort	21 (6f/15m)	66.3 ± 7.4	88 (33f/55m)	61.0 ± 11.9	NR	T1	ICV Correction	FreeSurfer	UK Brain Bank Criteria	MoCA HVLT, JLO, ANIM, LNS, SDMT
S. Danti 2015	Italy	Cross‐sectional study	18 (4f/14m)	66.5 ± 6.7	18 (3f/15m)	60.6 ± 9.0	1.5 T Siemens	T1, 3D MPRAGE	corrected	FreeSurfer	UPDRS	MDS PD‐MCI Level II
Dalaker 2011	Norway	Cross‐sectional study	11 (6f/5m)	67.5 ± 9.3	32 (11f/21m)	63.3 ± 10.2	1.5 T Philips	3D T1	TIV Correction	FreeSurfer	UPDRS	MMSE, MADRS
Apostolova 2010	USA	Cohort	8 (3f/5m)	78 ± 7.8	12 (6f/6m)	68.4 ± 6.8	1.5T Philips	T1, T2, FLAIR	Corrected	Manual + automated machine‐learning	UPDRS	MMSE

Abbreviations: f, female; FIRST, FMRIB's Integrated Registration and Segmentation Tool; GE, General Electric; ICV, intracranial volume; m, male; MADRS, Montgomery‐Asberg Depression Rating Scale, T1, T1 weight; MAGeT‐Brain, Multiple Automatically Generated Templates Brain; MDRS‐2, Mattis Dementia Rating Scale‐2; MDS‐UPDRS, Movement Disorder Society‐sponsored UPDRS revision; MMSE, Mini‐Mental State Examination; MoCA, Montreal Cognitive Assessment; NINDS/NINCDS, National Institute of Neurological Disorders and Stroke (criteria); NR, not reported; PD, Parkinson's disease; PD‐MCI, PD with mild cognitive impairment; PD‐NC, PD with no cognitive impairment; T2, T2‐weighted image; TIV, total intracranial volume; UPDRS, Unified Parkinson's Disease Rating Scale.

### 3.2 Quality Evaluation of Included Literature

The quality of the literature was assessed using the NOS method (Stang 2010), and the results of the quality assessment of the included literature are listed in Table S2.

### 3.3 Meta‐Analysis of Regional Volumetric Alterations

#### 3.3.1 Hippocampus

**FIGURE 2:**
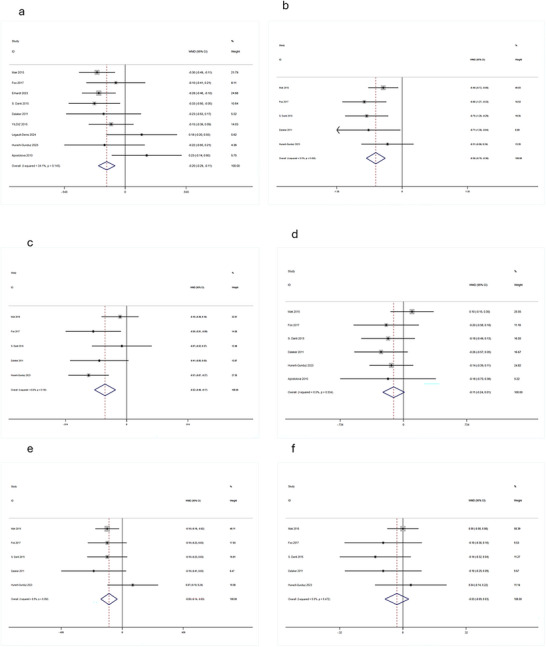
Forest plots of left subcortical structure volumes in PD‐MCI vs. PD‐NC. (a) Hippocampus, (b) thalamus, (c) putamen, (d) caudate, (e) amygdala, (f) globus pallidus. Forest plots show WMD (cm^3^) with 95% CI. Diamond: pooled effect size.

**FIGURE 3:**
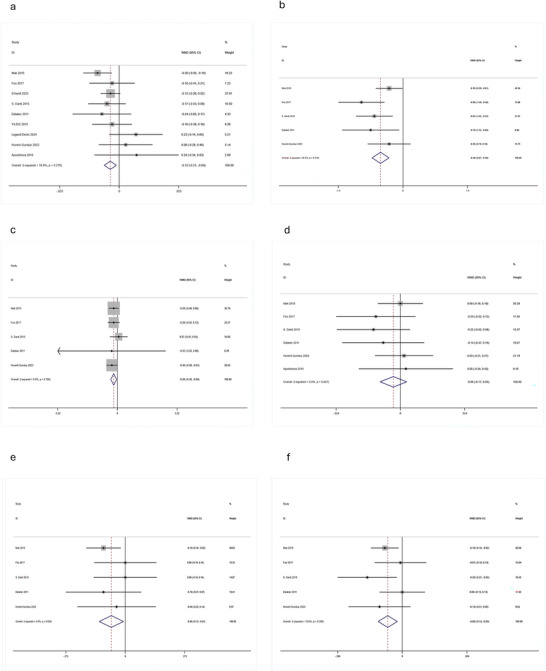
Forest plots of right subcortical structure volumes in PD‐MCI vs. PD‐NC. (a) Hippocampus, (b) thalamus, (c) putamen, (d) caudate, (e) amygdala, (f) globus pallidus. Forest plots show WMD (cm^3^) with 95% CI. Diamond: pooled effect size.

**FIGURE 4:**
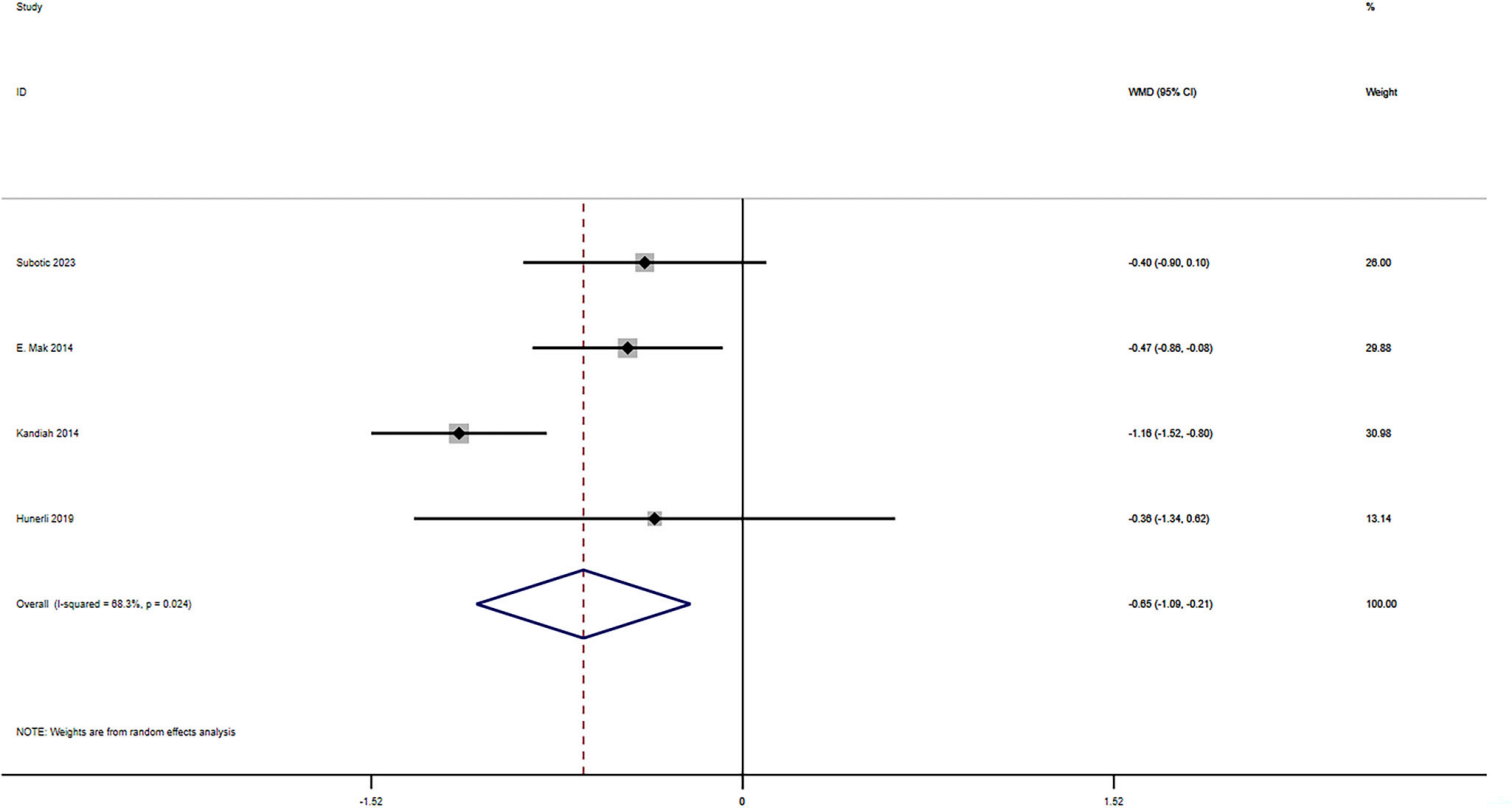
Forest plot of total hippocampal volume differences between PD‐MCI and PD‐NC groups. Pooled analysis demonstrates significant hippocampal atrophy in PD‐MCI WMD = –0.65 cm^3^, 95% CI: –1.09 to –0.21, *p* < 0.05. Heterogeneity *I* ^2^ = 68.3%.

Significant volume reductions in PD‐MCI were observed for total hippocampal volume (WMD = –0.65 cm^3^, 95% CI: [–1.09, –0.21]; *p* < 0.05) (Figure 4), left hippocampus (WMD = –0.20 cm^3^, 95% CI: [–0.29, –0.11]; *p* < 0.05) (Figure 2a) and right hippocampus (WMD = –0.12 cm^3^, 95% CI: [–0.21, –0.04]; *p* < 0.05) (Figure 3a ). Substantial heterogeneity was observed for the total hippocampal volume analysis (*I* ^2^ = 68.3%).

#### 3.3.2 Thalamus

Significant bilateral volume reductions were found (left: WMD = –0.56 cm^3^, 95% CI: [–0.76, –0.36]; *p* < 0.05; right: WMD = –0.49 cm^3^, 95% CI: [–0.67, –0.30]; *p* < 0.05) (Figures 2b and 3b).

#### 3.3.3 Putamen

Significant bilateral volume reductions were observed (left: WMD = –0.32 cm^3^, 95% CI: [–0.48, –0.17]; *p* < 0.05; right: WMD = –0.20 cm^3^, 95% CI: [–0.36, –0.04]; *p* < 0.05) (Figures 2c and 3c).

#### 3.3.4 Caudate Nucleus

Meta‐analysis revealed no statistically significant volume differences in PD‐MCI for either the left (WMD = –0.11 cm^3^, 95% CI: [–0.24, 0.01]; *p* > 0.05) or right caudate nucleus (WMD = –0.06 cm^3^, 95% CI: [–0.17, 0.05]; *p* > 0.05) (Figures 2d and 3d).

#### 3.3.5 Amygdala

Significant bilateral volume reductions were observed (left: WMD = –0.09 cm^3^, 95% CI: [–0.14, –0.03]; *p* < 0.05; right: WMD = –0.06 cm^3^, 95% CI: [–0.12, –0.01]; *p* < 0.05) (Figures 2e and 3e).

#### 3.3.6 Globus Pallidus

Significant volumetric alterations were observed in the right globus pallidus (WMD = –0.08 cm^3^, 95% CI: [–0.14, –0.03]; *p* < 0.05), while no significant change was found in the left globus pallidus (WMD = –0.03 cm^3^, 95% CI: [–0.09, 0.03]; *p* > 0.05) (Figures 2f and 3f).

### 3.4 Publication Bias

**FIGURE 5:**
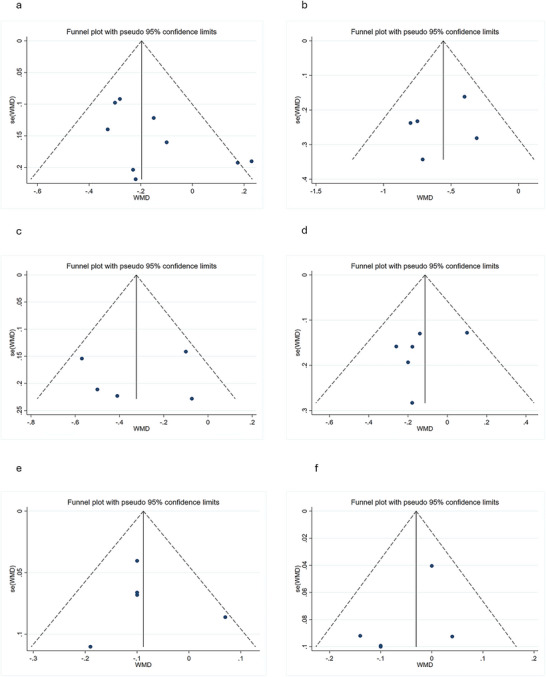
Funnel plots assessing publication bias for left hemisphere structures. (a) Hippocampus, (b) thalamus, (c) putamen, (d) caudate, (e) amygdala, (f) globus pallidus. Symmetric distribution suggests minimal bias, corroborating Table 2 statistical results.

**FIGURE 6:**
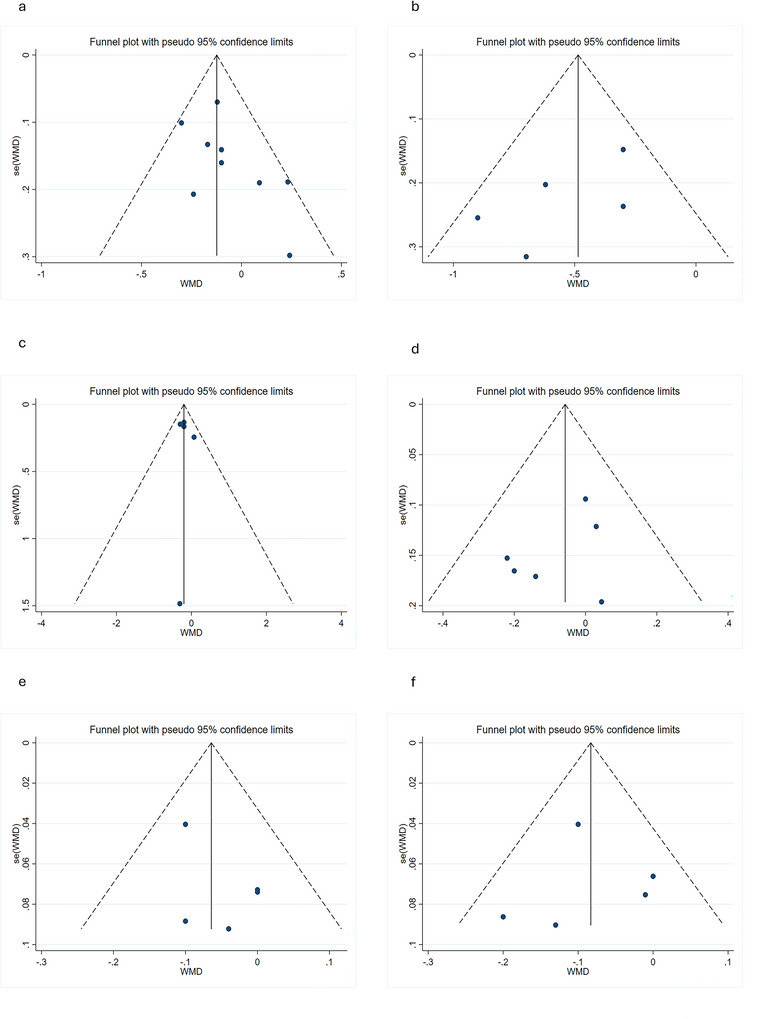
Funnel plots assessing publication bias for right hemisphere structures. (a) Hippocampus, (b) thalamus, (c) putamen, (d) caudate, (e) amygdala, (f) globus pallidus. Symmetric distribution suggests minimal bias, corroborating Table 2 statistical results.

Both visual assessment of funnel plots (e.g., Figures 5 and 6) and statistical analyses (Begg's and Egger's tests; Table 2) demonstrated no significant publication bias across the 13 included studies (all *p*‐values > 0.05).

**TABLE 2:** Assessment of publication bias for subcortical volumes in PD‐MCI.

Brain region	Begg's test		Egger's test	
	*Z*	*p*	*t*	*p*
L‐Hippocampus	1.36	0.175	2.13	0.071
R‐Hippocampus	1.36	0.175	1.57	0.160
L‐Thalamus	0.24	0.806	−0.61	0.587
R‐Thalamus	0.73	0.462	0.24	0.828
L‐Putamen	0.24	0.806	−0.19	0.863
R‐Putamen	−0.24	1.000	0.53	0.634
L‐Caudate	0.75	0.452	−1.05	0.355
R‐Caudate	0.00	1.000	−1.16	0.309
L‐Amygdala	0.24	0.806	0.27	0.804
R‐Amygdala	−0.24	1.000	1.34	0.272
L‐Globus pallidus	0.24	0.806	−1.44	0.244
R‐Globus pallidus	0.24	0.806	−0.08	0.942

*Note*: Results indicate no significant publication bias across all regions (all *p* > 0.05), supporting the robustness of meta‐analytic findings.

Abbreviations: L = left; PD‐MCI = Parkinson's disease with mild cognitive impairment; R = right.

### 3.5 Sensitivity Analysis

Leave‐one‐out sensitivity analysis confirmed robust volumetric reductions across key subcortical structures. In regions exhibiting significant atrophy in primary analysis—including the hippocampus, thalamus, putamen, amygdala, and right globus pallidus—pooled effect sizes remained statistically significant (WMD < 0; 95% CIs excluding zero) throughout sequential study exclusions. Hippocampal total volume reduction persisted (WMD: –0.73 to –0.44 cm^3^; Table S1; Figure S3) with bilateral subgroup atrophy (left: –0.22 to –0.17 cm^3^, Table S2; Figure S1a; right: –0.14 to –0.08 cm^3^, Table S3; Figure S2a). Similarly, thalamic atrophy maintained significance bilaterally (left: –0.66 to –0.50 cm^3^, Table S4; Figure S1b; right: –0.61 to –0.42 cm^3^, Table S5; Figure S2b), as did putaminal reductions (left: –0.43 to –0.23 cm^3^, Table S6; Figure S1c; right: –0.23 to –0.16 cm^3^, Table S7; Figure S2c). Amygdala vulnerability was consistently observed (left: –0.11 to –0.08 cm^3^, Table S10; Figure S1e; right: –0.07 to –0.06 cm^3^, Table S11; Figure S2e). Notably, while right globus pallidus atrophy persisted (WMD: –0.10 to –0.08 cm^3^; Table S13; Figure S2f), left globus pallidus volumes showed no significant change (WMD: –0.09 to 0.02 cm^3^, 95% CIs included zero; Table S12; Figure S1f). Crucially, structures initially non‐significant—specifically the bilateral caudate nucleus and left globus pallidus—retained null effects across all sensitivity analysis (Tables S8, S9, and S12; and Figures S1d, S2d, and S1f).

### 3.6 Meta‐Regression and Subgroup Analysis

Meta‐regression analysis revealed significant sources of heterogeneity in the left hippocampus, with segmentation tools (coefficient = 0.256, *p* = 0.044) and country (coefficient = –0.074, *p* = 0.047) showing statistically significant associations. In contrast, analysis of total hippocampal volume (*I* ^2^ = 68.3%) did not reveal any significant associations with any pre‐specified covariates (including segmentation tool, MRI field strength, scanning device, volume correction method, or country; all *p* > 0.20) (Table 3). Similarly, no significant covariates were observed in the other regions studied (thalamus, putamen, caudate nucleus, amygdala, globus pallidus) (all *p* > 0.05) (Tables S16).

**TABLE 3:** Meta‐regression of covariates for total hippocampal volume heterogeneity.

Brain region	Covariate	Coef.	*p*
Total hippocampus	Segmentation tool	−0.7166161	0.206
	Field strength	0.4593079	0.653
	Scanning device	−0.4193079	0.622
	Volume correction method	−0.3214705	0.263
	Country	0.7043056	0.321

*Note*: Partitioning tool, MRI equipment, and country did not significantly explain heterogeneity (all *p* > 0.20), suggesting unmeasured sources of variation.

Abbreviations: Coef = regression coefficient.

Subgroup analysis revealed that the type of segmentation tool significantly affected the effect size of the left hippocampal volume (between‐group *p* = 0.015). FreeSurfer reported a significant and consistent volume reduction (WMD = –0.27), while other tools did not detect significant changes (WMD = –0.03). This indicates that the difference in measurement tools is an important source of heterogeneity in this study (Figure S4).

## 4 Discussion

This meta‐analysis provides the first comprehensive quantitative synthesis of subcortical gray matter volume alterations in PD‐MCI, substantially extending previous research predominantly focused on hippocampal alterations (Yazdan Panah et al. 2023). Significant atrophy was observed within a distributed subcortical network comprising the hippocampus, thalamus, putamen, and amygdala in affected patients compared to PD‐NC controls. Importantly, methodological heterogeneity—particularly divergent segmentation tools (e.g., FreeSurfer vs. FSL‐FIRST)—emerged as the primary source of inter‐study variability, with meta‐regression confirming its dominant impact on left hippocampal volumetric differences. This broader structural alteration pattern, together with the identified methodological influences, distinguishes these findings from earlier, single‐structure–focused reports.

The hippocampal volume reduction corresponds to its established role in memory consolidation (Das et al. 2019; Grothe et al. 2016), providing a structural basis for episodic memory deficits characteristic of PD‐MCI. Concurrent thalamic degeneration likely disrupts multimodal information integration (Biesbroek et al. 2024; Schmitt et al. 2017), contributing to multi‐domain cognitive impairment. Putaminal atrophy reflects early involvement of cortico‐striatal‐thalamo‐cortical (CSTC) circuits critical for executive functioning (van Beilen and Leenders 2006). Amygdala reduction implicates limbic–emotional network disruption (Bigot et al. 2024), potentially explaining affective comorbidities such as anxiety and depression. In contrast, preserved caudate nucleus volumes suggest region‐specific vulnerability within the basal ganglia, indicating preferential targeting of posterior striatal (putamen) and limbic structures over associative striatal regions (Yoo et al. 2024).

A novel lateralized pattern was identified: significant atrophy occurred exclusively in the right globus pallidus, with no volumetric change in the left. This asymmetry implies lateralized dysfunction in circuits integrating motor and limbic functions, aligning with emerging evidence of right‐lateralized pathology in early PD‐MCI (Liu et al. 2024; Zhu et al. 2023). The combined pattern—putamen, amygdala, and right globus pallidus involvement with caudate sparing—differentiates PD‐MCI from Alzheimer's disease (AD) (Adler et al. 2018; Wu et al. 2025), offering a structural explanation for the prominence of executive dysfunction and affective symptoms over isolated amnestic deficits in PD‐MCI.

Subgroup analysis revealed that FreeSurfer consistently detected significant hippocampal atrophy, whereas other methods did not. Technical covariates (MRI field strength, scanner model, volume correction) showed no significant association with heterogeneity in any region. These findings underscore the urgent need for harmonized neuroimaging pipelines and adoption of robust segmentation protocols—preferably FreeSurfer in multi‐site studies—to enhance comparability and reduce methodological noise.

An important methodological consideration is the dynamic and non‐linear trajectory of neurodegeneration in Parkinson's disease. Brain atrophy does not progress at a constant rate but varies across disease stages, with potential early compensatory phases followed by accelerated decline. Because the included studies reported volumetric data at heterogeneous disease durations and primarily employed cross‐sectional designs, cross‐sectional pooling may introduce timepoint heterogeneity and stage‐mixed effects. Consequently, the pooled weighted mean differences reported here reflect disease‐stage–averaged differences rather than true longitudinal progression rates. These findings should therefore be interpreted as overall structural differences across mixed disease stages rather than annualized atrophy rates.

Several limitations warrant consideration. First, volumetric discrepancies stemming from heterogeneous segmentation methods remain a major constraint. Second, the predominance of cross‐sectional data limits causal inference; longitudinal studies are required to clarify the temporal sequence of atrophy and cognitive decline. Third, variability in PD‐MCI diagnostic criteria underscores the importance of adopting standardized Movement Disorder Society guidelines. Fourth, insufficient reporting of clinical covariates (e.g., dopaminergic medication effects, disease duration stratification) hampers mechanistic interpretation. Finally, heterogeneity in disease duration across studies may have influenced effect size estimates, as volumetric differences were measured at different stages of disease progression.

Future research should prioritize methodological harmonization and longitudinal validation. First, standardizing segmentation protocols is essential to reduce inter‐study variability; evidence suggests that harmonized pipelines (e.g., FreeSurfer) may enhance sensitivity to hippocampal atrophy and improve cross‐cohort comparability. Second, given the dynamic and non‐linear progression of neurodegeneration in Parkinson's disease, longitudinal studies with standardized follow‐up intervals are needed to estimate annualized atrophy rates and stage‐specific structural trajectories, thereby addressing temporal heterogeneity and strengthening structure–cognition prediction models for dementia conversion. Third, integrating multimodal imaging with comprehensive emotional and behavioral assessments will clarify the clinical relevance of amygdala degeneration and limbic network disruption in PD‐MCI.

## 5 Conclusion

In conclusion, PD‐MCI exhibits a distinct subcortical atrophy signature involving limbic (hippocampus, amygdala) and striato‐thalamic networks, with right globus pallidus atrophy emerging as a novel lateralized biomarker. These findings underscore the necessity of network‐based imaging paradigms over single‐structure approaches. Translation to clinical practice requires: (1) harmonized MRI protocols to minimize methodological variability; (2) longitudinal validation of atrophy trajectories; and (3) integration with molecular imaging to dissect comorbid pathologies.

## Author Contributions

**Jinhua Hu**: methodology, investigation, writing – original draft. **Peng Lei**: methodology, investigation, writing – original draft. **Jupeng Zhang**: investigation, formal analysis, data curation, validation. **Qi Wu**: conceptualization, methodology, investigation, visualization, software, writing – review and editing. **Zhihao Zhang**: data curation, investigation, validation, formal analysis. **Xiqi Zhu**: project administration, funding acquisition, supervision, validation, writing – review and editing. **Baosheng Li**: conceptualization, writing – review and editing, supervision. All authors contributed to the interpretation of results, revised the manuscript critically for important intellectual content, approved the final version to be published, and agreed to be accountable for the integrity and accuracy of the work.

## Funding

This study was financially supported by the National Natural Science Foundation of China (Grant No. 82460226), the Natural Science Foundation of Guangxi Autonomous Region (Grant No. 2023GXNSFAA026383, 2025GXNSFHA069156, 2025GXNSFBA069280).

## Ethics Statement

This study utilized publicly available aggregated data and did not involve new human or animal participants; therefore, ethical approval was not required according to institutional policy.

## Conflicts of Interest

There are no potential conflicts of interest or personal relationships that could affect the research results reported in this article.

## Supporting information




**Supplementary Information**: brb371410‐sup‐0001‐SuppMat.docx

## Data Availability

The data supporting this meta‐analysis are available from the published articles cited in the manuscript. Extracted datasets and analysis scripts can be obtained from the corresponding author upon reasonable request.
