# Supplementary material for: Structural Volumetric Alterations in Parkinson's Disease With Mild Cognitive Impairment
Source: Brain Behav. 2026 Apr 27;16(5):e71410. doi: 10.1002/brb3.71410 (PMC13118392; doi:10.1002/brb3.71410)
Supplement: Supplementary file 1 — Supplementary Information: brb371410‐sup‐0001‐SuppMat.docx [file BRB3-16-e71410-s001.docx]

**Search Strategy**

**Publication of search results is limited to the period from inception to June 2025**

**1. The search formula for retrieving PubMed is as follows:**

(("Magnetic Resonance Imaging"[Mesh]) OR ((((((((((((((((((((((((((((((((((((((((Imaging, Multimodal) OR (Imaging, NMR)) OR (Zeugmatography)) OR (NMR Imaging)) OR (Tomography, MR)) OR (Steady-State Free Precession MRI)) OR (Steady State Free Precession MRI)) OR (NMR Tomography)) OR (Tomography, NMR)) OR (MR Tomography)) OR (Tomography, Proton Spin)) OR (Proton Spin Tomography)) OR (Magnetization Transfer Contrast Imaging)) OR (fMRI)) OR (Magnetic Resonance Imaging, Functional)) OR (MRI, Functional)) OR (Functional MRI)) OR (Functional MRIs)) OR (MRIs, Functional)) OR (Functional Magnetic Resonance Imaging)) OR (MRI Scans)) OR (MRI Scan)) OR (Scan, MRI)) OR (Scans, MRI)) OR (Imaging, Chemical Shift)) OR (Chemical Shift Imagings)) OR (Imagings, Chemical Shift)) OR (Shift Imaging, Chemical)) OR (Shift Imagings, Chemical)) OR (Chemical Shift Imaging)) OR (Spin Echo Imaging)) OR (Echo Imaging, Spin)) OR (Echo Imagings, Spin)) OR (Imaging, Spin Echo)) OR (Imagings, Spin Echo)) OR (Spin Echo Imagings)) OR (Magnetic Resonance Image)) OR (Image, Magnetic Resonance)) OR (Magnetic Resonance Images)) OR (Resonance Image, Magnetic))) AND ((((Brain Volume) OR ((((("Hippocampus"[Mesh]) OR ((((((((((((((((Ammon's Horn) OR (Ammons Horn)) OR (Horn, Ammon's)) OR (Cornu Ammonis)) OR (Ammon Horn)) OR (Horn, Ammon)) OR (Hippocampus Proper)) OR (Hippocampus Propers)) OR (Proper, Hippocampus)) OR (Propers, Hippocampus)) OR (Subiculum)) OR (Subiculums)) OR (Hippocampal Formation)) OR (Formation, Hippocampal)) OR (Formations, Hippocampal)) OR (Hippocampal Formations))) OR (("Atrophy"[Mesh]) OR (Atrophies))) OR (((((((((Matters, White) OR (Matter, White)) OR (White Matters)) OR (Cerebellar White Matter)) OR (Cerebellar White Matters)) OR (Matter, Cerebellar White)) OR (Matters, Cerebellar White)) OR (White Matter, Cerebellar)) OR (White Matters, Cerebellar))) OR (("Gray Matter"[Mesh]) OR (((((((((((((((((((Gray Matters) OR (Matter, Gray)) OR (Matters, Gray)) OR (Grey Matter)) OR (Grey Matters)) OR (Matter, Grey)) OR (Matters, Grey)) OR (Cerebellar Gray Matter)) OR (Cerebellar Gray Matters)) OR (Gray Matter, Cerebellar)) OR (Gray Matters, Cerebellar)) OR (Matter, Cerebellar Gray)) OR (Matters, Cerebellar Gray)) OR (Cerebellar Grey Matter)) OR (Cerebellar Grey Matters)) OR (Grey Matter, Cerebellar)) OR (Grey Matters, Cerebellar)) OR (Matter, Cerebellar Grey)) OR (Matters, Cerebellar Grey))))) AND (("Cognitive Dysfunction"[Mesh]) OR (((((((((((((((((((((((((Cognitive Dysfunctions) OR (Dysfunction, Cognitive)) OR (Dysfunctions, Cognitive)) OR (Cognitive Disorder)) OR (Cognitive Disorders)) OR (Disorder, Cognitive)) OR (Disorders, Cognitive)) OR (Cognitive Impairments)) OR (Cognitive Impairment)) OR (Impairment, Cognitive)) OR (Impairments, Cognitive)) OR (Mild Cognitive Impairment)) OR (Cognitive Impairment, Mild)) OR (Cognitive Impairments, Mild)) OR (Impairment, Mild Cognitive)) OR (Impairments, Mild Cognitive)) OR (Mild Cognitive Impairments)) OR (Cognitive Decline)) OR (Cognitive Declines)) OR (Decline, Cognitive)) OR (Declines, Cognitive)) OR (Mental Deterioration)) OR (Deterioration, Mental)) OR (Deteriorations, Mental)) OR (Mental Deteriorations)))) AND (("Parkinson Disease"[Mesh]) OR (((((((((((Idiopathic Parkinson Disease) OR (Idiopathic Parkinson's Disease)) OR (Lewy Body Parkinson Disease)) OR (Lewy Body Parkinson's Disease)) OR (Paralysis Agitans)) OR (Parkinson Disease, Idiopathic)) OR (Parkinson's Disease)) OR (Parkinson's Disease, Idiopathic)) OR (Parkinson's Disease, Lewy Body)) OR (Primary Parkinsonism)) OR (Parkinsonism, Primary))))

**2. The search formula for retrieving Embase is as follows:**

(('mild cognitive impairment'/exp OR 'amnestic mild cognitive impairment':ab,ti OR 'mild cognitive impairment':ab,ti)) AND(('gray matter'/exp OR 'brain gray matter':ab,ti OR 'brain grey matter':ab,ti OR 'cerebellar gray matter':ab,ti OR 'cerebellum gray matter':ab,ti OR 'grey matter':ab,ti OR 'spinal cord gray matter':ab,ti OR 'spinal gray matter':ab,ti OR 'substantia grisea':ab,ti OR 'superficial gray matter':ab,ti OR 'gray matter':ab,ti) OR ('white matter'/exp OR 'brain white matter':ab,ti OR 'cerebellar white matter':ab,ti OR 'cerebellum white matter':ab,ti OR 'cerebral white matter':ab,ti OR 'spinal cord white matter':ab,ti OR 'spinal white matter':ab,ti OR 'substantia alba':ab,ti OR 'substantia alba cerebelli':ab,ti OR 'substantia alba medullae spinalis':ab,ti OR 'white matter':ab,ti) OR ('atrophy'/exp OR panatrophy:ab,ti OR atrophy:ab,ti)) AND('hippocampus'/exp OR 'ammon horn':ab,ti OR 'cornu ammonis':ab,ti OR hipocampus:ab,ti OR 'hippocampal efferent':ab,ti OR 'hippocampal formation':ab,ti OR 'hippocampal region':ab,ti OR hypocampus:ab,ti OR 'hyppocampus pes':ab,ti OR hippocampus:ab,ti) AND('brain size'/exp OR 'brain volume':ab,ti OR 'brain size':ab,ti) AND

('parkinson disease'/exp OR 'idiopathic parkinsonism':ab,ti OR 'lewy bodies of parkinson disease':ab,ti OR 'lewy bodies of parkinson`s disease':ab,ti OR 'lewy bodies of parkinsons disease':ab,ti OR 'lewy body parkinson disease':ab,ti OR 'lewy body parkinson`s disease':ab,ti OR 'lewy body parkinsons disease':ab,ti OR 'paralysis agitans':ab,ti OR 'parkinson dementia complex':ab,ti OR 'parkinson`s disease':ab,ti OR 'parkinsons disease':ab,ti OR 'primary parkinsonism':ab,ti OR 'parkinson disease':ab,ti)

**3. The search formula for searching Cochrane is as follows:**

1 MeSH descriptor: [Radiation, Ionizing] explode all trees

#2 MeSH descriptor: [Radiologists] explode all trees

#3 (Radiologists):ti,ab,kw OR (Radiography):ti,ab,kw OR (radiographers):ti,ab,kw OR (radiology staff):ti,ab,kw OR (radiology workers):ti,ab,kw OR (Radiation workers):ti,ab,kw OR (radiation field workers):ti,ab,kw OR (Medical Radiographers):ti,ab,kw OR (Industrial Irradiation Workers):ti,ab,kw OR (Radiation-Exposed Workers):ti,ab,kw OR (Radiation, Ionizing):ti,ab,kw OR (Low-Dose Ionizing Radiation):ti,ab,kw OR (Low-Dose Radiation):ti,ab,kw OR (Low Radiation Doses):ti,ab,kw OR (low-dose medical radiation exposure):ti,ab,kw OR (radiation exposure):ti,ab,kw OR (occupational exposure of X-Ray):ti,ab,kw

#4 #1 OR #2 OR #3

#5 MeSH descriptor: [Blood Cell Count] explode all trees

#6 MeSH descriptor: [Erythrocyte Count] explode all trees

#7 MeSH descriptor: [Leukocyte Count] explode all trees

#8 MeSH descriptor: [Erythrocyte Indices] explode all trees

#9 MeSH descriptor: [Hemoglobinometry] explode all trees

#10 (blood cells):ti,ab,kw OR (Blood Cell Count):ti,ab,kw OR (Leukocyte Count):ti,ab,kw OR (Leukocyte Counts):ti,ab,kw OR (Leukocyte Number):ti,ab,kw OR (Leukocyte Numbers):ti,ab,kw OR (Complete Blood Count):ti,ab,kw OR (Complete Blood Counts):ti,ab,kw OR (CBC):ti,ab,kw OR (White Blood Cell Count):ti,ab,kw OR (Differential Leukocyte Count):ti,ab,kw OR (hematological parameters):ti,ab,kw OR (hematological indices):ti,ab,kw OR (Biological Effects):ti,ab,kw OR (hematological index):ti,ab,kw OR (hematological change):ti,ab,kw OR (Hematological Factors):ti,ab,kw OR (WBC):ti,ab,kw OR (white blood cell):ti,ab,kw OR (white blood cells):ti,ab,kw OR (hemoglobin):ti,ab,kw OR (Hemoglobinometry):ti,ab,kw OR (Red Blood Cell):ti,ab,kw OR (Red Blood Cells):ti,ab,kw OR (Erythrocyte Counts):ti,ab,kw OR (Erythrocyte Number):ti,ab,kw OR (Erythrocyte Numbers):ti,ab,kw OR (Erythrocyte Indices):ti,ab,kw OR (Red Cell Indices):ti,ab,kw OR (Red Cell Indexes):ti,ab,kw OR (Erythrocyte Index):ti,ab,kw OR (Erythrocyte Indexes):ti,ab,kw OR (Platelet Count):ti,ab,kw OR (Platelet Counts):ti,ab,kw OR (Platelet Number):ti,ab,kw OR (Platelet Numbers):ti,ab,kw OR (Blood Platelet Number):ti,ab,kw OR (Blood Platelet Numbers):ti,ab,kw OR (Blood Platelet Count):ti,ab,kw OR (Blood Platelet Counts):ti,ab,kw

#11 #5 OR #6 OR #7 OR #8 OR #9 OR #10

#12 (prospective cohort study):ti,ab,kw OR (the control group):ti,ab,kw OR (controls):ti,ab,kw OR (case-control)

#13 #4 AND #11 AND #12 with Publication Year from 2024 to 2025, in Trials

**4. The search formula for searching Web of Science is as follows:**

Cognitive Dysfunction (Topic) or Cognitive Dysfunctions (Topic) or Dysfunction, Cognitive (Topic) or Dysfunctions, Cognitive (Topic) or Cognitive Disorder (Topic) or Cognitive Disorders (Topic) or Disorder, Cognitive (Topic) or Disorders, Cognitive (Topic) or Cognitive Impairments (Topic) or Cognitive Impairment (Topic) or Impairment, Cognitive (Topic) or Impairments, Cognitive (Topic) or Mild Cognitive Impairment (Topic) or Cognitive Impairment, Mild (Topic) or Cognitive Impairments, Mild (Topic) or Impairment, Mild Cognitive (Topic) or Impairments, Mild Cognitive (Topic) or Mild Cognitive Impairments (Topic) or Cognitive Decline (Topic) or Cognitive Declines (Topic) or Decline, Cognitive (Topic) or Declines, Cognitive (Topic) or Mental Deterioration (Topic) or Deterioration, Mental (Topic) or Deteriorations, Mental (Topic) or Mental Deteriorations (Topic) and Preprint Citation Index (Exclude – Database) AND Parkinson disease (Topic) or idiopathic parkinsonism (Topic) or Lewy bodies of Parkinson disease (Topic) or Lewy bodies of Parkinson's disease (Topic) or Lewy bodies of Parkinsons disease (Topic) or Lewy body Parkinson disease (Topic) or Lewy body Parkinson's disease (Topic) or Lewy body Parkinsons disease (Topic) or paralysis agitans (Topic) or Parkinson dementia complex (Topic) or Parkinson's disease (Topic) or Parkinsons disease (Topic) or primary parkinsonism (Topic) or Parkinson disease (Topic) and Preprint Citation Index (Exclude – Database) AND Brain volume (Topic) or volume (Topic) and Preprint Citation Index (Exclude – Database) AND （Hippocampus (Topic) or Ammon's Horn (Topic) or Ammons Horn (Topic) or Horn, Ammon's (Topic) or Cornu Ammonis (Topic) or Ammon Horn (Topic) or Horn, Ammon (Topic) or Hippocampus Proper (Topic) or Hippocampus Propers (Topic) or Proper, Hippocampus (Topic) or Propers, Hippocampus (Topic) or Subiculum (Topic) or Subiculums (Topic) or Hippocampal Formation (Topic) or Formation, Hippocampal (Topic) or Formations, Hippocampal (Topic) or Hippocampal Formations (Topic) and Preprint Citation Index (Exclude – Database) OR Atrophy (Topic) or Atrophies (Topic) and Preprint Citation Index (Exclude – Database) OR White Matter (Topic) or Matters, White (Topic) or Matter, White (Topic) or White Matters (Topic) or Cerebellar White Matter (Topic) or Cerebellar White Matters (Topic) or Matter, Cerebellar White (Topic) or Matters, Cerebellar White (Topic) or White Matter, Cerebellar (Topic) or White Matters, Cerebellar (Topic) and Preprint Citation Index (Exclude – Database) OR Gray Matter (Topic) or Gray Matters (Topic) or Matter, Gray (Topic) or Matters, Gray (Topic) or Grey Matter (Topic) or Grey Matters (Topic) or Matter, Grey (Topic) or Matters, Grey (Topic) or Cerebellar Gray Matter (Topic) or Cerebellar Gray Matters (Topic) or Gray Matter, Cerebellar (Topic) or Gray Matters, Cerebellar (Topic) or Matter, Cerebellar Gray (Topic) or Matters, Cerebellar Gray (Topic) or Cerebellar Grey Matter (Topic) or Cerebellar Grey Matters (Topic) or Grey Matter, Cerebellar (Topic) or Grey Matters, Cerebellar (Topic) or Matter, Cerebellar Grey (Topic) and Preprint Citation Index (Exclude – Database)）

| (a) | (b) |
| --- | --- |
|  |  |
| (c) | (d) |
|  |  |
| (e) | (f) |
|  |  |

Figure S1 Sensitivity Analysis of Left Subcortical Volumes

Forest plots displaying weighted mean differences (WMD) and heterogeneity (I²) for volumetric comparisons between Parkinson's disease with mild cognitive impairment (PD-MCI) and cognitively normal Parkinson's disease (PD-NC) groups. Regions include: (a) left hippocampus, (b) left thalamus, (c) left putamen, (d) left caudate nucleus, (e) left amygdala, (f) left globus pallidus. Diamonds represent pooled effect sizes; horizontal lines indicate 95% confidence intervals. Sensitivity analyses confirm robustness of significant atrophy in hippocampus, thalamus, putamen, and amygdala (all WMD < 0; 95% CIs exclude zero), while caudate nucleus and globus pallidus show non-significant differences (95% CIs include zero).

| (a) | (b) |
| --- | --- |
|  |  |
| (c) | (d) |
|  |  |
| (e) | (f) |
|  |  |

Figure S 2 Sensitivity Analysis of Right Subcortical Volumes

Forest plots illustrating weighted mean differences (WMD) and heterogeneity (I²) for volumetric differences (PD-MCI vs. PD-NC). Regions include: (a) right hippocampus, (b) right thalamus, (c) right putamen, (d) right caudate nucleus, (e) right amygdala, (f) right globus pallidus. Results demonstrate stable significant atrophy in hippocampus, thalamus, putamen, amygdala, and right globus pallidus (all WMD < 0; 95% CIs exclude zero), with preserved volumes in caudate nucleus (95% CIs include zero).

Figure S 3 Sensitivity Analysis of Total Hippocampal Volume

Forest plot demonstrating high heterogeneity (I² = 68.3%) in total hippocampal volume comparisons between PD-MCI and PD-NC groups. Pooled WMD = -0.65 cm³ (95% CI: -1.09 to -0.21), consistent with significant atrophy reported in primary meta-analysis. Subgroup analyses revealed no significant moderators (segmentation tools, MRI field strengths, geographic regions; all P > 0.20), indicating unexplained heterogeneity possibly due to unmeasured clinical covariates.


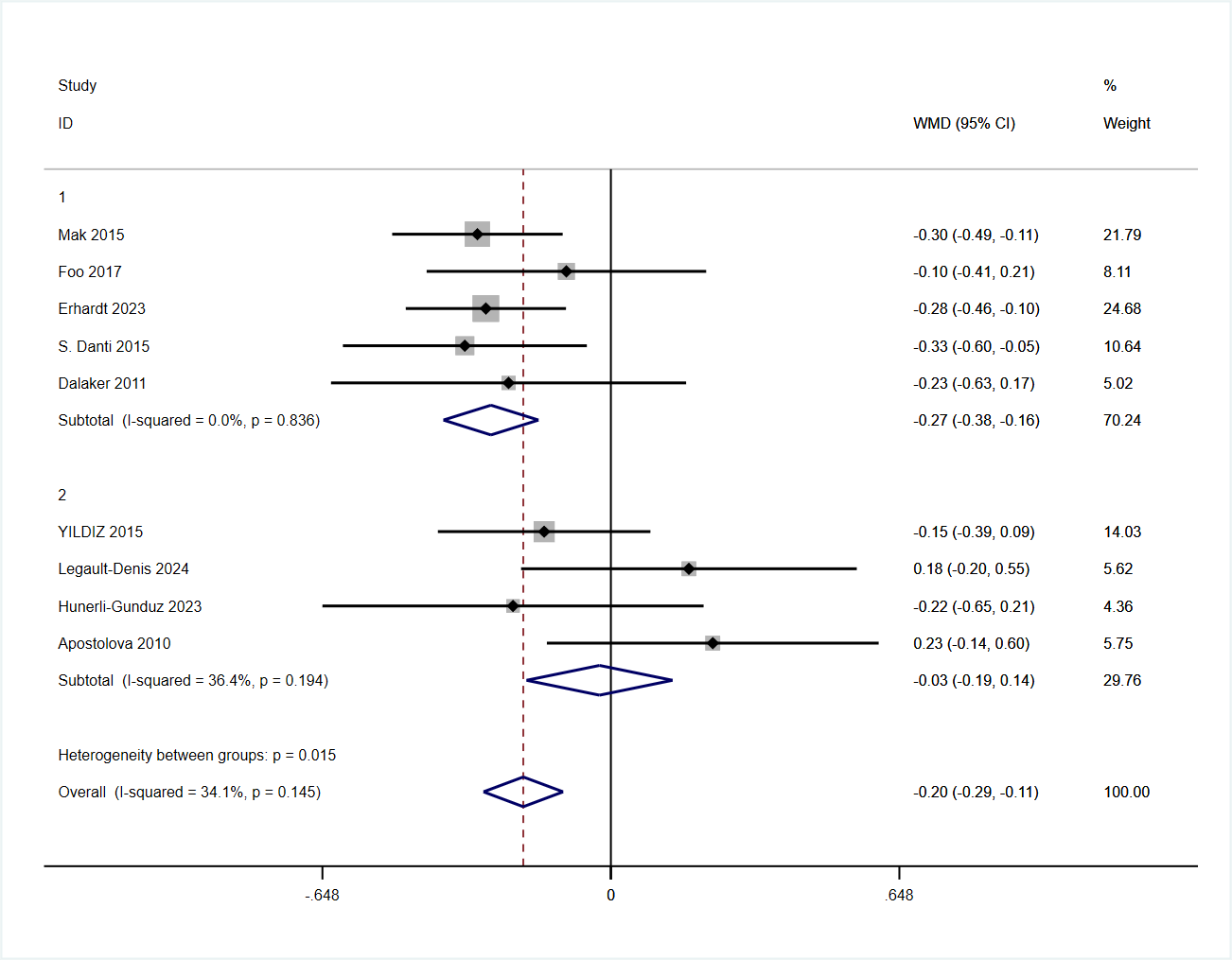


Figure S 4 Forest Plot of Left Hippocampal Volume Subgroup Analysis Stratified by Segmentation Tool

The analysis compared FreeSurfer (Subgroup 1) versus other segmentation tools (Subgroup 2). Diamonds represent pooled effect sizes (WMD). Between-group heterogeneity was statistically significant (p = 0.015).

Table S 1 Total hippocampus volume sensitivity analysis

| Study omitted | Estimate | [95% Conf. | Interval] |
| --- | --- | --- | --- |
| Subotic 2023 | -.7309139 | -1.2872153 | -.17461245 |
| E. Mak 2014 | -.71016244 | -1.3106088 | -.1097161 |
| Kandiah 2014 | -.43601207 | -.72858129 | -.14344285 |
| Hunerli 2019 | -.69292605 | -1.1941618 | -.19169029 |
| Combined | -.65110166 | -1.0887728 | -.2134305 |

Leave-one-out sensitivity analysis of total hippocampal volume. Pooled weighted mean differences (WMD) and 95% confidence intervals remained statistically significant (WMD range: -0.73 to -0.44 cm³) after sequential exclusion of individual studies, confirming the robustness of hippocampal atrophy in PD-MCI (vs. PD-NC).

Table S 2 Left hippocampus volume sensitivity analysis

| Study omitted | Estimate | [95% Conf. | Interval] |
| --- | --- | --- | --- |
| Mak 2015 | -.16817963 | -.26932202 | -.06703724 |
| Foo 2017 | -.20545492 | -.29876263 | -.1121472 |
| Erhardt 2023 | -.16935758 | -.27241771 | -.06629746 |
| S. Danti 2015 | -.18126019 | -.2758833 | -.08663707 |
| Dalaker 2011 | -.19515545 | -.28693599 | -.1033749 |
| YILDIZ 2015 | -.20455757 | -.30102335 | -.1080918 |
| Legault-Denis 2024 | -.21908177 | -.31115258 | -.12701096 |
| Hunerli-Gunduz 2023 | -.19585323 | -.2873145 | -.10439196 |
| Apostolova 2010 | -.22290365 | -.31503876 | -.13076853 |
| Combined | -.19690581 | -.28635143 | -.1074602 |

Sensitivity analysis of left hippocampal volume. Consistent significant atrophy was observed across all exclusions (WMD range: -0.22 to -0.17 cm³; all 95% CIs exclude zero), supporting the stability of left hippocampal volume reduction in PD-MCI.

Table S 3 Right hippocampus volume sensitivity analysis

| Study omitted | Estimate | [95% Conf. | Interval] |
| --- | --- | --- | --- |
| Mak 2015 | -.08419598 | -.17761879 | .00922683 |
| Foo 2017 | -.12534785 | -.21306405 | -.03763166 |
| Erhardt 2023 | -.12504988 | -.2322679 | -.01783186 |
| S. Danti 2015 | -.11814746 | -.20744869 | -.02884622 |
| Dalaker 2011 | -.11823849 | -.20461523 | -.03186176 |
| YILDIZ 2015 | -.125943 | -.21468296 | -.03720303 |
| Legault-Denis 2024 | -.14308138 | -.22985819 | -.05630456 |
| Hunerli-Gunduz 2023 | -.13508997 | -.22183447 | -.04834547 |
| Apostolova 2010 | -.13130153 | -.21668378 | -.04591929 |
| Combined | -.12351456 | -.20799917 | -.03902995 |

Sensitivity analysis of right hippocampal volume. Pooled estimates remained significant upon sequential study removal (WMD range: -0.14 to -0.08 cm³), reinforcing right-lateralized hippocampal vulnerability in PD-MCI.

Table S 4 Left Thalamus Volume Sensitivity Analysis

| Study omitted | Estimate | [95% Conf. | Interval] |
| --- | --- | --- | --- |
| Mak 2015 | -.66240213 | -.92112585 | -.4036784 |
| Foo 2017 | -.50218352 | -.72416186 | -.28020519 |
| S. Danti 2015 | -.51133326 | -.73444085 | -.28822566 |
| Dalaker 2011 | -.54247919 | -.75238368 | -.33257469 |
| Hunerli-Gunduz 2023 | -.59499417 | -.81005281 | -.37993553 |
| Combined | -.55736777 | -.75772752 | -.35700802 |

Leave-one-out sensitivity analysis for left thalamic volume. Robust volumetric reductions persisted (WMD range: -0.66 to -0.50 cm³; all p<0.05), indicating high reliability of left thalamic atrophy findings.

Table S 5 Right thalamus volume sensitivity analysis

| Study omitted | Estimate | [95% Conf. | Interval] |
| --- | --- | --- | --- |
| Mak 2015 | -.61153901 | -.84985435 | -.37322366 |
| Foo 2017 | -.42066578 | -.61869927 | -.2226323 |
| S. Danti 2015 | -.44905848 | -.65677292 | -.24134404 |
| Dalaker 2011 | -.46503884 | -.65785497 | -.2722227 |
| Hunerli-Gunduz 2023 | -.5205506 | -.72106701 | -.3200342 |
| Combined | -.48586251 | -.66993618 | -.30178883 |

Sensitivity analysis of right thalamic volume. Significant bilateral atrophy was maintained across exclusions (WMD range: -0.61 to -0.42 cm³), with no study disproportionately influencing the pooled effect.

Table S 6 Left putamen volume sensitivity analysis

| Study omitted | Estimate | [95% Conf. | Interval] |
| --- | --- | --- | --- |
| Mak 2015 | -.43189488 | -.62423677 | -.23955299 |
| Foo 2017 | -.29393773 | -.46489811 | -.12297735 |
| S. Danti 2015 | -.35981442 | -.52872209 | -.19090675 |
| Dalaker 2011 | -.3110494 | -.4805168 | -.141582 |
| Hunerli-Gunduz 2023 | -.23136849 | -.4167455 | -.04599148 |
| Combined | -.32397956 | -.48198709 | -.16597203 |

Sensitivity analysis for left putamen volume. Pooled WMD remained significant (range: -0.43 to -0.23 cm³) and directionally consistent after sequential exclusion, confirming striatal vulnerability in PD-MCI.

Table S 7 Right putamen volume sensitivity analysis

| Study omitted | Estimate | [95% Conf. | Interval] |
| --- | --- | --- | --- |
| Mak 2015 | -.20132702 | -.39774393 | -.00491012 |
| Foo 2017 | -.20111491 | -.38115064 | -.02107918 |
| S. Danti 2015 | -.23348516 | -.40013238 | -.06683794 |
| Dalaker 2011 | -.20053169 | -.35815492 | -.04290846 |
| Hunerli-Gunduz 2023 | -.15927036 | -.34678481 | .02824409 |
| Combined | -.2008521 | -.35824449 | -.04345971 |

Sensitivity analysis of right putamen volume. Effect estimates retained statistical significance (WMD range: -0.23 to -0.16 cm³) across all exclusions, supporting bilateral putaminal degeneration.

Table S 8 Left caudate volume sensitivity analysis

| Study omitted | Estimate | [95% Conf. | Interval] |
| --- | --- | --- | --- |
| Mak 2015 | -.18708174 | -.33391521 | -.04024827 |
| Foo 2017 | -.10286012 | -.23729338 | .03157314 |
| S. Danti 2015 | -.1009676 | -.23965898 | .03772372 |
| Dalaker 2011 | -.08445322 | -.22324434 | .05433789 |
| Hunerli-Gunduz 2023 | -.10505274 | -.25116494 | .04105946 |
| Apostolova 2010 | -.11013205 | -.24026453 | .02000043 |
| Combined | -.11372514 | -.2404174 | .01296712 |

Leave-one-out sensitivity analysis for left caudate nucleus. Non-significant pooled estimates persisted (WMD range: -0.19 to -0.08 cm³; all 95% CIs include zero), affirming absence of volumetric differences between PD-MCI and PD-NC.

Table S 9 Right caudate volume sensitivity analysis

| Study omitted | Estimate | [95% Conf. | Interval] |
| --- | --- | --- | --- |
| Mak 2015 | -.08828464 | -.22429007 | .04772078 |
| Foo 2017 | -.03877312 | -.15501144 | .0774652 |
| S. Danti 2015 | -.03199637 | -.14955551 | .08556277 |
| Dalaker 2011 | -.04724465 | -.16301128 | .06852198 |
| Hunerli-Gunduz 2023 | -.08056326 | -.20381156 | .04268504 |
| Apostolova 2010 | -.06614897 | -.18028934 | .04799139 |
| Combined | -.05714041 | -.16655753 | .05227672 |

Sensitivity analysis of the right caudate nucleus. Consistently non-significant results (WMD range: -0.09 to -0.03 cm³) were observed across exclusions, reinforcing caudate preservation in PD-MCI.

Table S 10 Left amygdala volume sensitivity analysis

| Study omitted | Estimate | [95% Conf. | Interval] |
| --- | --- | --- | --- |
| Mak 2015 | -.07654816 | -.15278054 | -.00031578 |
| Foo 2017 | -.08517129 | -.14578945 | -.02455312 |
| S. Danti 2015 | -.08536562 | -.14561039 | -.02512085 |
| Dalaker 2011 | -.08076715 | -.13754776 | -.02398653 |
| Hunerli-Gunduz 2023 | -.10649682 | -.16456742 | -.04842622 |
| Combined | -.08783071 | -.14274478 | -.03291664 |

Sensitivity analysis for left amygdala volume. Statistically significant atrophy (WMD range: -0.11 to -0.08 cm³) persisted upon sequential exclusion, highlighting robust limbic involvement.

Table S 11 Right amygdala volume sensitivity analysis

| Study omitted | Estimate | [95% Conf. | Interval] |
| --- | --- | --- | --- |
| Mak 2015 | -.02837005 | -.10726763 | .05052753 |
| Foo 2017 | -.07566071 | -.13639561 | -.0149258 |
| S. Danti 2015 | -.07524817 | -.13581727 | -.01467907 |
| Dalaker 2011 | -.05988626 | -.11892851 | -.000844 |
| Hunerli-Gunduz 2023 | -.06660657 | -.12537419 | -.00783896 |
| Combined | -.06406093 | -.11994651 | -.00817535 |

Sensitivity analysis of right amygdala volume. Effect sizes remained significant (WMD range: -0.07 to -0.06 cm³) with all 95% CIs excluding zero, confirming bilateral amygdala degeneration.

Table S 12 Left pallidus volume sensitivity analysis

| Study omitted | Estimate | [95% Conf. | Interval] |
| --- | --- | --- | --- |
| Mak 2015 | -.07335685 | -.16715235 | 0.02043864 |
| Foo 2017 | -.02320278 | -.08680895 | 0.04040339 |
| S. Danti 2015 | -.01662101 | -.08084678 | 0.04760477 |
| Dalaker 2011 | -.02307815 | -.08673591 | 0.04057962 |
| Hunerli-Gunduz 2023 | -.03935796 | -.10353711 | 0.0248212 |
| Combined | -.03052025 | -.10353711 | 0.02997979 |

Leave-one-out sensitivity analysis for left globus pallidus. Non-significant pooled estimates (WMD range: -0.04 to -0.01 cm³) were unchanged across exclusions, consistent with primary meta-analysis results.

Table S 13 Right pallidus volume sensitivity analysis

| Study omitted | Estimate | [95% Conf. | Interval] |
| --- | --- | --- | --- |
| Mak 2015 | -.0674586 | -.14364008 | .00872287 |
| Foo 2017 | -.0947906 | -.15392908 | -.03565212 |
| S. Danti 2015 | -.06931938 | -.12736072 | -.01127804 |
| Dalaker 2011 | -.10124893 | -.16184149 | -.04065637 |
| Hunerli-Gunduz 2023 | -.07811255 | -.13585468 | -.02037042 |
| Combined | -.08310324 | -.13799827 | -.0282082 |

Sensitivity analysis of right globus pallidus. Significant right-lateralized atrophy was maintained (WMD range: -0.10 to -0.08 cm³), supporting asymmetric subcortical vulnerability patterns.

Table S 14 Reporting Formats and Units for Subcortical Volumetric Data Across Included Studies

| Author  (year) | Organization | Values |
| --- | --- | --- |
| Mak 2015 | cm^3^ | mean ±SD |
| Foo 2017 | cm^3^ | mean ±SD |
| Erhardt 2023 | mm^3^ | median [Q1, Q3] |
| S. Danti 2015 | mm^3^ | mean ±SD |
| Dalaker 2011 | cm^3^ | mean ±SD |
| YILDIZ 2015 | cm^3^ | mean ±SD |
| Legault-Denis 2024 | mm^3^ | mean ±SD |
| Hunerli-Gunduz 2023 | cm^3^ | mean ±SD |
| Apostolova 2010 | mm^3^ | mean ±SD |
| Subotic 2023 | cm^3^ | mean ±SD |
| E. Mak 2014 | cm^3^ | mean ±SD |
| Kandiah 2014 | cm^3^ | mean ±SD |
| Hunerli 2019 | cm^3^ | mean ±SD |

Summary of measurement units (cm³ or mm³) and data presentation formats (mean ± SD or median [IQR]) for subcortical volumes in 12 studies. Note: Unit inconsistency was adjusted by applying scaling factors (1 cm³ = 1000 mm³) prior to meta-analysis.

Table S 15 Quality Assessment of Included Studies Using Newcastle-Ottawa Scale

|  | Selection | | | | Comparability | | Outcome | | | Score |
| --- | --- | --- | --- | --- | --- | --- | --- | --- | --- | --- |
| Study | Representative | Selection of non-exposed | Ascertainment of exposure | Outcome not present at start | Comparability on most important factors | Comparability on other risk factors | Outcome assessment | Long enough follow-up (median ≥ 1 year) | Adequacy of follow-up (completeness) |  |
| YILDIZ 2015 | * | * | * | - | * | - | * | - | - | 6 |
| Subotic 2023 | * | * | * | * | * | * | * | - | * | 8 |
| Mak 2015 | * | * | * | * | * | * | * | * | - | 8 |
| E. Mak 2014 | * | * | * | * | * | - | * | * | - | 7 |
| Legault-Denis 2024 | * | * | * | * | * | - | * | - | * | 7 |
| Kandiah 2014 | * | * | * | * | * | - | - | * | * | 7 |
| Hunerli-Gunduz 2023 | * | * | * | * | * | * | * | - | * | 8 |
| Hunerli 2019 | * | * | * | * | * | * | * | - | * | 8 |
| Foo 2017 | * | * | * | * | * | - | - | * | - | 6 |
| Erhardt 2023 | * | * | * | * | * | * | * | * | * | 9 |
| S. Danti 2015 | * | * | * | - | * | * | * | * | - | 7 |
| Dalaker 2011 | * | * | * | - | * | * | * | * | * | 8 |
| Apostolova 2010 | * | * | * | * | * | * | * | * | * | 9 |

Evaluation of study quality across three domains: Selection (max 4 stars), Comparability (max 2 stars), and Outcome (max 3 stars). Total scores range from 6 to 9 stars, with higher scores indicating superior methodological rigor. Studies by Erhardt et al. (2023) and Apostolova et al. (2010) achieved the highest score (9/9).

Table S 16 Meta-Regression of Covariates Explaining Heterogeneity in Subcortical Volumes

| Brain Region | Covariate | Coef. | P |
| --- | --- | --- | --- |
| Left Hippocampus | Segmentation tool | 0.256066 | 0.044 |
|  | Field strength | -0.1001766 | 0.270 |
|  | Scanning device | -0.0434894 | 0.422 |
|  | Volume correction method | 0.2350102 | 0.192 |
|  | country | -0.0739379 | 0.047 |
| right Hippocampus | Segmentation tool | 0.1866139 | 0.402 |
|  | Field strength | 0.0186224 | 0.920 |
|  | Scanning device | -0.0375771 | 0.810 |
|  | Volume correction method | .1474515 | 0.419 |
|  | country | -.0282561 | 0.686 |
| L-Thalamus | Segmentation tool | 0.4260531 | 0.363 |
|  | Field strength | -0.1941695 | 0.531 |
|  | Scanning device | -0.0052754 | 0.991 |

Table S 16 continued

| Brain Region | Covariate | Coef. | P |
| --- | --- | --- | --- |

|  | Volume correction method | -0.3916652 | 0.351 |
| --- | --- | --- | --- |
|  | country | 0.0113025 | 0.912 |
| R-Thalamus | Segmentation tool | 0.3512178 | 0.490 |
|  | Scanning device | 0.1258084 | 0.765 |
|  | Volume correction method | -0.5304329 | 0.266 |
|  | country | 0.0368884 | 0.718 |
| L-Putamen | Segmentation tool | -0.326209 | 0.405 |
|  | Field strength | 0.0179675 | 0.953 |
|  | Scanning device | 0.3214184 | 0.611 |
|  | Volume correction method | -0.162723 | 0.718 |
|  | country | 0.0396871 | 0.752 |
| R-Putamen | Segmentation tool | 0.01 | 0.996 |
|  | Field strength | -0.11 | 0.953 |

Table S 16 continued

| Brain Region | Covariate | Coef. | P |
| --- | --- | --- | --- |

|  | Scanning device | 0.37871 | 0.843 |
| --- | --- | --- | --- |
|  | Volume correction method | 0.1455211 | 0.481 |
|  | country | 0.067515 | 0.426 |
| L-Caudate | Segmentation tool | 0.0723878 | 0.687 |
|  | Field strength | -0.2278262 | 0.237 |
|  | Scanning device | 0.0463089 | 0.857 |
|  | country | 0.0006163 | 0.991 |
| R-Caudate | Segmentation tool | 0.1741507 | 0.475 |
|  | Field strength | -0.091179 | 0.678 |
|  | Scanning device | -0.08003 | 0.760 |
|  | Volume correction method | -0.1442814 | 0.314 |
|  | country | -0.0126244 | 0.771 |
| L-Amygdala | Segmentation tool | 0.2556399 | 0.205 |
|  | Field strength | -0.0856399 | 0.527 |

Table S 16 continued

| Brain Region | Covariate | Coef. | P |
| --- | --- | --- | --- |

|  | Scanning device | -0.0145744 | 0.982 |
| --- | --- | --- | --- |
|  | Volume correction method | -0.0872371 | 0.495 |
|  | country | -0.025034 | 0.520 |
| R-Amygdala | Segmentation tool | 0.0254403 | 0.812 |
|  | Field strength | 0.0035059 | 0.967 |
|  | Scanning device | 0.071224 | 0.548 |
|  | Volume correction method | 0.0775359 | 0.331 |
| L-Globus pallidus | Segmentation tool | 0.14 | 0.490 |
|  | Field strength | -0.0859719 | 0.566 |
|  | Scanning device | -0.04 | 0.817 |
|  | Volume correction method | -0.1144695 | 0.262 |
|  | country | -0.0054831 | 0.850 |

Table S 16 continue

| Brain Region | Covariate | Coef. | P |
| --- | --- | --- | --- |
| R-Globus pallidus | Segmentation tool | -0.13 | 0.462 |
|  | Field strength | 0.0776733 | 0.506 |
|  | Scanning device | -0.2 | 0.325 |
|  | Volume correction method | 0.060087 | 0.610 |
|  | country | -0.0078097 | 0.805 |

Association between pre-specified covariates and volumetric differences (PD-MCI vs. PD-NC) across 11 brain regions. Significant findings: Left hippocampus: Segmentation tool (β = 0.256, *P* = 0.044) and country (β = -0.074, *P* = 0.047). Total hippocampus: No significant covariates (*P* >0.20). *Abbreviations*: Coef., regression coefficient; Partitioning tool, segmentation method (FreeSurfer/FSL-FIRST/Other).
